# Association of Nursing Home Exposure to Hurricane-Related Inundation With Emergency Preparedness

**DOI:** 10.1001/jamanetworkopen.2022.49937

**Published:** 2023-01-06

**Authors:** Natalia Festa, Kaitlin F. Throgmorton, Nora Heaphy, Maureen Canavan, Thomas M. Gill

**Affiliations:** 1 Department of Internal Medicine, Yale School of Medicine, New Haven, Connecticut; 2 National Clinician Scholars Program at Yale University, New Haven, Connecticut; 3 Harvey Cushing/John Hay Whitney Medical Library, School of Medicine, Yale University, New Haven, Connecticut; 4 Department of Ecology and Evolutionary Biology, Yale University, New Haven, Connecticut; 5 Department of Internal Medicine, Cancer Outcomes and Public Policy and Effectiveness Research, Yale School of Medicine, New Haven, Connecticut

## Abstract

**Importance:**

Whether US nursing homes are well prepared for exposure to hurricane-related inundation is uncertain.

**Objectives:**

To estimate the prevalence of nursing homes exposed to hurricane-related inundation and evaluate whether exposed facilities are more likely to meet Centers for Medicare & Medicaid Services (CMS) emergency preparedness standards.

**Design, Setting, and Participants:**

This cross-sectional study included CMS-certified nursing homes in Coastal Atlantic and Gulf Coast states from January 1, 2017, to December 31, 2019. The prevalence of facilities exposed to at least 2 feet of hurricane-related inundation used models from the National Hurricane Center across coastal areas overseen by 5 CMS regional offices: New England, New York metropolitan area, Mid-Atlantic region, Southeast and Eastern Gulf Coast, and Western Gulf Coast. Critical emergency preparedness deficiencies cited during CMS life safety code inspections were identified.

**Main Outcomes and Measures:**

The analysis used generalized estimating equations with binomial and negative binomial distributions to evaluate associations between exposure status and the presence and number of critical emergency preparedness deficiencies. Regionally stratified associations (odds ratios [ORs]) and rate ratios [RRs]) with 95% CIs, adjusted for state-level fixed effects and nursing home characteristics, were reported.

**Results:**

Of 5914 nursing homes, 617 (10.4%) were at risk of inundation exposure, and 1763 (29.8%) had a critical emergency preparedness deficiency. Exposed facilities were less likely to be rural, were larger, and had similar CMS health inspection, quality, and staffing ratings compared with unexposed facilities. Exposure was positively associated with the presence and number of emergency preparedness deficiencies for the nursing homes within the Mid-Atlantic region (adjusted OR, 1.91 [95% CI, 1.15-3.20]; adjusted RR, 2.51 [95% CI, 1.41-4.47]). Conversely, exposure was negatively associated with the number of emergency preparedness deficiencies among facilities within the Western Gulf Coast (aRR, 0.55 [95% CI, 0.36-0.86]). The associations for the number of emergency preparedness deficiencies remained after correction for multiple comparisons.

**Conclusions and Relevance:**

The findings of this cross-sectional study suggest that the association between exposure to hurricane-related inundation and nursing home emergency preparedness differs considerably across the Coastal Atlantic and Gulf regulatory regions. These findings further suggest that there may be opportunities to reduce regional heterogeneity and improve the alignment of nursing home emergency preparedness with surrounding environmental risks.

## Key Points

**Question:**

Are nursing homes exposed to potential hurricane-related inundation more likely to meet Centers for Medicare & Medicaid Services criteria for adequate emergency preparedness?

**Findings:**

In this cross-sectional study of 5914 nursing homes, a higher prevalence of emergency preparedness deficiencies among nursing homes exposed to hurricane-related inundation in the Mid-Atlantic region was observed. Exposure status remained positively associated with the presence and number of emergency preparedness deficiencies after adjustment for facility characteristics, with the converse for facilities within the Western Gulf Coast.

**Meaning:**

These findings suggest opportunities to reduce regional heterogeneity and improve the alignment of nursing home emergency preparedness with surrounding environmental risks.

## Teaser

This cross-sectional study estimates the prevalence of nursing homes that are subject to severe hurricane-related inundation within the Coastal Atlantic and Gulf Coast states and evaluates whether exposure status is associated with adherence to emergency preparedness standards across regulatory regions.

## Introduction

Nursing homes’ emergency preparedness is vital due to projected increases in the frequency with which coastal communities are exposed to severe weather events.^1,2,3,4^ An estimated 1.5 million older persons are long-term care residents of nursing homes, while 27% of hospitalized older persons are discharged to these settings for postacute care.^5^ The advanced age, medical complexity, and custodial needs of this population impose a differential risk of morbidity and mortality due to environmental hazards.^6,7^ The adverse outcomes incurred by nursing home residents following Hurricanes Katrina and Rita highlight this population’s unique susceptibility to environmental exposures and the importance of adequate emergency preparedness.^8,9^ Despite the risks borne by nursing home residents, variation in their exposure to potential environmental hazards is not well characterized. It is also uncertain whether nursing homes at increased risk of exposure are adequately prepared to safeguard residents upon encountering environmental hazards.

The paucity of information regarding nursing homes’ preparedness for local environmental hazards may be attributed, in part, to the inherent complexity of characterizing small-area variation in environmental exposures. Because environmental exposures vary across communities, the Centers for Medicare & Medicaid Services (CMS) has advised an all-hazards approach to emergency preparedness.^10,11^ This requires that facility administrators and staff assess, document, and plan for potential hazards, including severe weather events, that are expected to impact their geographical region.^10,11^ Emergency preparedness is then audited by state survey agencies, which are overseen by CMS regional offices.^10,11^

Considering their competing responsibilities,^12^ nursing home management and staff and the superstructure of state survey agencies and CMS regional offices may not be well positioned to appraise and prepare for potential environmental hazards. Therefore, the CMS encourages partnerships with municipal agencies to ensure that nursing homes are embedded within community disaster planning and response.^10,13^ Despite this recommendation, nursing home integration into community disaster preparedness efforts remains variable.^14,15^ Prior assessments of nursing home emergency preparedness, moreover, have identified widespread deficiencies in critical competencies that include appropriate specification of evacuation routes and preparedness to shelter residents in place.^2,3,16^ These preventable deficiencies heighten the risk of resident morbidity and mortality following exposure to severe weather events.^6,7,8,17^

In this study, we estimate the prevalence of nursing homes that are subject to severe hurricane-related inundation within Coastal Atlantic and Gulf Coast states. We then evaluate whether exposure status is associated with adherence to emergency preparedness standards by nursing homes across regulatory regions overseen by 5 CMS regional offices. The results of this study should inform practices and policies designed to align nursing home emergency preparedness with the hurricane-related inundation risk to which facilities are exposed.

## Methods

This cross-sectional study followed the Strengthening the Reporting of Observational Studies in Epidemiology (STROBE) reporting guideline. This research was deemed exempt from review and informed consent by the Yale University Institutional Review Board under 45 CFR 46.104.

### Data Sources and Sampling Approach

We utilized the CMS Provider Information Catalog to identify certified nursing homes within states that are at risk of exposure to hurricane-related inundation along the Coastal Atlantic and Gulf Coast regions as per the National Hurricane Center (NHC) (n = 6167).^18,19^ We evaluated facilities’ emergency preparedness deficiencies based on inspections from January 1, 2017, to December 31, 2019. We removed nursing homes with missing facility characteristics or inspection data during the observation window (n = 253), leading to a final analytic sample of 5914 facilities.

### Exposure Definition

We defined exposed nursing homes as those at risk of at least 2 feet of inundation during Saffir-Simpson Hurricane Wind Scale category 1 to 5 hurricanes as per the NHC storm surge hazard maps.^19,20^ Our primary exposure threshold was informed by the US National Weather Service determination that roads are impassable at inundation heights above 18 to 24 inches,^21^ which would preclude the safe ground evacuation of residents and transport of essential medications or subsistence provisions. The NHC data do not project inundation within levee systems because its predictions are constrained to land that is normally dry.^19^ We classified the 46 nursing homes within leveed areas as exposed because levee systems are both subject to failure and concentrated in areas with disproportionate risk of severe flooding.^22^

The methods used by the NHC to model inundation have been described previously.^19^ Briefly, the NHC storm surge hazard maps aggregate maximum inundation heights, assuming a high-tide initial water level.^19^ Storm surge events occur when the height of water generated by a storm rises above that of normal astronomical tides.^19^ *Inundation*, the height of water above the ground surface, is estimated by subtracting the height of land elevation from that of the storm surge.^19^ The NHC uses the Sea, Lake, and Overland Surges from Hurricanes (SLOSH) model to simulate a representative sample of hypothetical storms, resulting in 2 data products.^19^ The first, maximum envelopes of water, represents the maximum inundation resulting from tens of thousands of hypothetical storms, with varying meteorological assumptions.^19^ The second represents the maximum composite value across the maximum envelopes of water.^19^

### Outcome Definition

We aggregated emergency preparedness deficiencies using CMS life safety code audits over 3 years from January 1, 2017, to December 31, 2019.^23^ From the 252 potential emergency preparedness deficiencies, we selected a subset deemed to be most critical in accordance with prior literature and emergency preparedness guidance (eTable 1 in Supplement 1).^16,24,25^ These deficiencies subsume competencies that are essential to safely sheltering residents in place and, when necessary, evacuating them. We defined a primary dichotomous outcome, classifying whether facilities were cited for any critical emergency preparedness deficiency during the observation window. We also defined a secondary count outcome, summing the number of qualifying critical emergency preparedness deficiencies per inspection per facility.

### Facility Characteristics

We used CMS facility identification numbers to link active nursing homes to facility characteristics. The facility characteristics included size, ownership status, and CMS 5-star quality ratings for the staffing, health inspection, and quality domains (greater number of stars indicates better rating). We used LTCFocus (Long-Term Care Focus) data to link lagged (2018) indicators of the payer mix and quantified the percentage of residents within each facility primarily insured by Medicaid.^26^ We also used information from the Health Resources and Services Administration, which used 2010 US Census information to designate rural areas.^27^

### Statistical Analysis

We first estimated the percentage of nursing homes at risk of exposure to at least 2 feet of hurricane-related inundation using their geocoded addresses. We described facility characteristics for the full sample and compared these characteristics among facilities according to their inundation exposure status. We also assessed the prevalence of critical emergency preparedness deficiencies across the nursing homes within each CMS regional office designation (described hereinafter).

We evaluated associations between location within an inundation-exposed area and emergency preparedness deficiencies (presence and count) using generalized estimating equations with binomial and negative binomial distributions, respectively, clustered by facility identification numbers, with robust standard errors. To address potential confounding, the models were adjusted for facility characteristics.

We stratified our analysis across the 5 CMS regional offices overseeing state survey agencies along the Coastal Atlantic and Gulf Coast. Regional office 1 of the CMS oversees New England (Connecticut, Maine, Massachusetts, New Hampshire, and Rhode Island); regional office 2, the New York metropolitan area (New York and New Jersey); regional office 3, the Mid-Atlantic region (Delaware, Maryland, and Virginia); regional office 4, the Southeast and Eastern Gulf Coast (Alabama, Florida, Georgia, Mississippi, North Carolina, and South Carolina); and regional office 6, the Western Gulf Coast (Louisiana and Texas). Regional office 5 was not evaluated because it oversees Midwestern states. To adjust for state-level variation in inspection practices and patterns,^28,29,30^ we included fixed effects for state within each stratified model. We used the Benjamini-Krieger-Yekutelli correction to account for multiple comparisons.^31^ In sensitivity analyses, we reran the models for the 5 regional office strata after increasing the severity of the inundation exposure threshold to at least 4 feet and at least 6 feet. We conducted analyses using ArcGIS Pro (Esri), Python, version 3 (Python.org), and Stata, version 17 (StataCorp LLC). Two-sided *P* < .05 indicated statistical significance.

## Results

**Figure 1.**
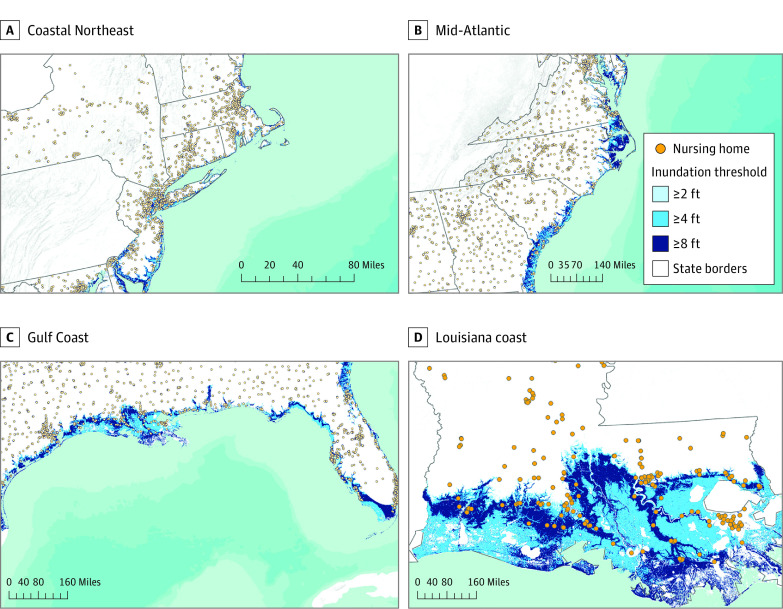
Nursing Home Exposure to Hurricane-Related Inundation Along the Atlantic and Gulf Coasts The geocoded locations of Centers for Medicare & Medicaid–certified nursing homes are displayed as of November 2021. Nursing home locations are superimposed on an adaptation of a National Hurricane Center map that predicts the maximum hurricane-related inundation in coastal areas. Three progressively severe categories of inundation are displayed for categories 1 through 5 hurricanes (≥2, ≥4, ≥6 feet). The Coastal Northeast includes Connecticut, Maine, Massachusetts, New Hampshire, New Jersey, New York, and Rhode Island; the Mid-Atlantic, Delaware, Maryland, and Virginia; and the Gulf Coast, Alabama, Florida, Georgia, Louisiana, Mississippi, North Carolina, South Carolina, and Texas.

Figure 1 displays the geocoded locations of the 5914 nursing homes relative to areas that are susceptible to hurricane-related inundation. A total of 617 nursing homes (10.4%) were at risk of exposure to at least 2 feet of hurricane-related inundation, and 1763 (29.8%) had a critical emergency preparedness deficiency. As shown in Table 1, fewer exposed than unexposed facilities were located within rural areas. Most facilities, exposed and unexposed, were proprietary. Exposed facilities were somewhat more likely than unexposed facilities to have higher CMS health inspection ratings, whereas quality and staffing ratings were balanced across the 2 groups. Compared with unexposed facilities, exposed facilities had a greater number of certified beds and lower Medicaid share. eTable 2 in Supplement 1 displays the number and percentage of nursing homes at risk of exposure to increasingly severe inundation thresholds, ranging from 2 to 6 feet, by CMS regional office. The Southeast and Eastern Gulf Coast had the highest percentage of nursing homes located in areas at risk of exposure to inundation of at least 2 feet (289 of 2103 [13.7%), at least 4 feet (253 of 2103 [12.0%]), and at least 6 feet (225 of 2103 [10.7%]).

**Table 1.** Sample Characteristics by Exposure to Hurricane-Related Inundation^a^

Characteristic	Facility type^b^			*P* value^b^
	Overall (N = 5914)	Unexposed (n = 5297)	Exposed (n = 617)	
Rurality	1203 (20.3)	1187 (22.4)	16 (2.6)	<.001
Proprietary ownership	4388 (74.2)	3940 (74.4)	448 (72.6)	.34
Health inspection rating, No. of stars^c^				
1	1136 (19.3)	1026 (19.5)	110 (17.9)	.10
2	1387 (23.6)	1247 (23.7)	140 (22.8)	
3	1349 (23.0)	1195 (22.7)	154 (25.1)	
4	1397 (23.8)	1266 (24.1)	131 (21.4)	
5	604 (10.3)	526 (10.0)	78 (12.7)	
Quality rating, No. of stars^c^				
1	400 (6.8)	351 (6.7)	49 (8.0)	.30
2	850 (14.5)	777 (14.8)	73 (11.9)	
3	1265 (21.6)	1133 (21.6)	132 (21.6)	
4	1549 (26.5)	1387 (26.4)	162 (26.5)	
5	1791 (30.6)	1596 (30.4)	195 (31.9)	
Staffing rating, No. of stars^c^				
1	1084 (18.6)	961 (18.4)	123 (20.2)	.11
2	1513 (25.9)	1379 (26.4)	134 (22.0)	
3	1449 (24.8)	1302 (24.9)	147 (24.1)	
4	1200 (20.6)	1067 (20.4)	133 (21.8)	
5	590 (10.1)	518 (9.9)	72 (11.8)	
Medicaid share, mean (SD), %	61.7 (22.8)	61.8 (22.8)	60.1 (23.9)	.01
No. of inspections, 2017-2020, mean (SD)	2.36 (0.59)	2.37 (0.59)	2.29 (0.57)	<.001
Total No. of beds, mean (SD)	121.7 (67.5)	120.3 (66.1)	133.6 (77.4)	<.001

^a^ Exposed nursing homes are located within an area subject to at least 2 feet of hurricane-related inundation during categories 1 to 5 hurricanes. Unless otherwise indicated, data are expressed as No. (%) of facilities. Percentages have been rounded and may not total 100. Owing to missing data for some facilities, numbers in each category may sum less than totals in column headings.

^b^ Differences between exposed and unexposed nursing homes were tested using 2-sided tests for categorical characteristics and unpaired *t* tests for continuous characteristics.

^c^ The Centers for Medicare & Medicaid Services 5-star rating system is a publicly reported measure of nursing home quality, with a higher number of stars indicating better quality across 3 domains (staffing, quality, and health inspections).

Table 2 provides the prevalence and number of critical emergency preparedness deficiencies by CMS regional office for the unexposed and exposed facilities. The New York metropolitan area had the highest total percentage of facilities with a critical emergency preparedness deficiency (346 of 967 [35.8%]), with little difference between the unexposed and exposed facilities. The Mid-Atlantic region had the highest percentage (16 of 30 [53.3%]) of exposed nursing homes with a critical emergency preparedness deficiency and the greatest mean (SD) number of deficiencies per exposed facility (4.0 [5.8]). The Western Gulf Coast had the lowest percentage (18 of 160 [11.3%]) of exposed nursing homes with a critical emergency preparedness deficiency and the smallest mean (SD) number of deficiencies per exposed facility (0.2 [0.8]).

**Table 2.** Summary of Emergency Preparedness Deficiencies by CMS Regional Office and Potential Inundation Exposure^a^

Coastal states by CMS regional office	No. of nursing homes	Nursing homes with critical deficiency, No. (%)	No. of critical deficiencies per nursing home, mean (SD)	No. of critical deficiencies, range
New England				
Total	819	251 (30.6)	1.2 (2.8)	0-28
Unexposed	784	241 (30.7)	1.2 (2.9)	0-28
Exposed	35	10 (28.6)	1.0 (1.9)	0-6
New York metropolitan area				
Total	967	346 (35.8)	0.9 (1.8)	0-15
Unexposed	864	308 (35.6)	0.9 (1.8)	0-15
Exposed	103	38 (36.9)	0.8 (1.6)	0-9
Mid-Atlantic region				
Total	565	102 (18.1)	1.1 (3.5)	0-25
Unexposed	535	86 (16.1)	1.0 (3.3)	0-25
Exposed	30	16 (53.3)	4.0 (5.8)	0-22
Southeast and Eastern Gulf Coast				
Total	2103	552 (26.2)	0.7 (1.8)	0-20
Unexposed	1814	436 (24.0)	0.6 (1.7)	0-20
Exposed	289	116 (40.1)	1.3 (2.3)	0-12
Western Gulf Coast				
Total	1460	512 (35.1)	1.1 (2.4)	0-20
Unexposed	1300	436 (33.5)	1.2 (2.5)	0-20
Exposed	160	18 (11.3)	0.2 (0.8)	0-6

Abbreviation: CMS, Centers for Medicare & Medicaid Services.

^a^ Exposed nursing homes are located within an area subject to at least 2 feet of hurricane-related inundation during categories 1 to 5 hurricanes.

The most common emergency preparedness deficiencies among the 5914 nursing homes related to appropriate emergency preparedness training and procedures, as well as the establishment and maintenance of an emergency preparedness program (eTable 1 in Supplement 1). The most common deficiencies varied across regional offices (eTable 3 in Supplement 1). For New England, New York metropolitan area, and Mid-Atlantic facilities, the most prevalent deficiencies related to appropriate emergency preparedness training and procedures, as well as securing adequate subsistence provisions of medication, food, and potable water. For facilities in the Southeast and Eastern Gulf Coast and Western Gulf Coast, prevalent deficiencies included the failure to establish and maintain an emergency preparedness program, as well as failure to conduct appropriate risk assessment using an all-hazards approach.

**Figure 2.**
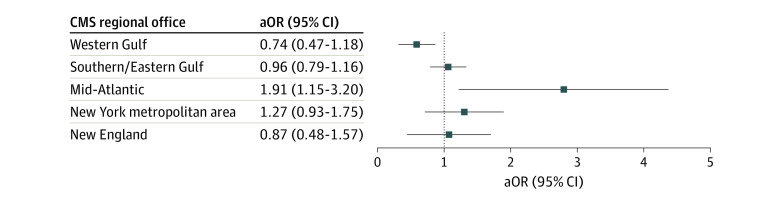
Association Between Exposure to Hurricane-Related Inundation and the Presence of an Emergency Preparedness Deficiency The adjusted odds ratios (aORs) of a critical emergency preparedness deficiency among facilities located within an exposed area (subject to ≥2 feet of hurricane-related inundation) are displayed and stratified by Centers for Medicare & Medicaid (CMS) regional office. The reported associations are adjusted for rurality, proprietary ownership, facility size, Medicaid share, and CMS 5-star quality and staffing ratings (where a higher number of stars indicates better quality across 3 domains [staffing, quality, and health inspections]).

As shown in Figure 2, exposure status was positively associated with the adjusted odds of a critical emergency preparedness deficiency for the nursing homes within the Mid-Atlantic region (adjusted odds ratio [aOR], 1.91 [95% CI, 1.15-3.20]). The nursing homes within the New York metropolitan area followed a similar pattern but results were not significant (aOR, 1.27 [95% CI, 0.93-1.75]). Exposure was negatively associated with critical emergency preparedness deficiencies for the facilities within New England (aOR, 0.87 [95% CI, 0.48-1.57]), the Southeast and Eastern Gulf Coast (aOR, 0.96 [95% CI, 0.79-1.16]), and the Western Gulf Coast (aOR, 0.74 [95% CI, 0.47-1.18]) but results were not significant. The adjusted association for the Mid-Atlantic region did not remain after correction for multiple comparisons.

**Figure 3.**
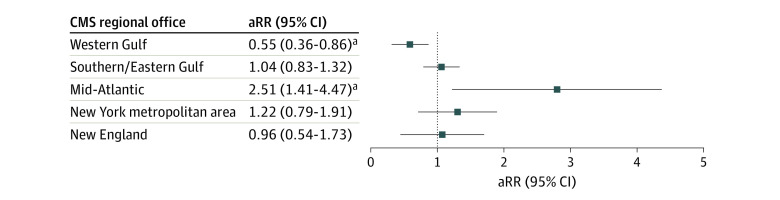
Association Between Exposure to Hurricane-Related Inundation and the Count of Emergency Preparedness Deficiencies The adjusted rate ratio (aRR) of critical emergency preparedness deficiencies among facilities located within an exposed area (subject to ≥2 feet of hurricane-related inundation) are displayed and stratified by Centers for Medicare & Medicaid (CMS) regional office. The reported associations are adjusted for rurality, proprietary ownership, facility size, Medicaid share, and CMS 5-star quality and staffing ratings (where a higher number of stars indicates better quality across 3 domains [staffing, quality, and health inspections]). ^a^Indicates a statistically significant association after Benjamin-Krieger-Yekutieli false discovery rate correction.

As shown in Figure 3, exposure was positively associated with the number of critical emergency preparedness deficiencies for nursing homes within the Mid-Atlantic region (adjusted rate ratio [aRR], 2.51 [95% CI, 1.41-4.47]). We observed a similar pattern for nursing homes within the New York metropolitan area (aRR, 1.22 [95% CI, 0.79-1.91]) and the Southeast and Eastern Gulf Coast (aRR, 1.04 [95% CI, 0.83-1.32]), but results were not significant. Exposure was negatively associated with the number of critical emergency preparedness deficiencies for nursing homes within the Western Gulf Coast (aRR, 0.55 [95% CI, 0.36-0.86]). We observed a similar pattern for nursing homes within New England (aRR, 0.96 [95% CI, 0.54-1.73]) but results were not significant. The adjusted associations for the Mid-Atlantic region and Western Gulf Coast remained after correction for multiple comparisons.

### Sensitivity Analysis

The magnitude of associations was either similar or did not follow a consistent pattern when the inundation threshold was increased to at least 4 feet and at least 6 feet (eTable 4 in the Supplement). Inundation of at least 4 feet was associated with emergency preparedness deficiencies in the Mid-Atlantic region (aOR, 1.68 [95% CI, 1.00-2.83]).

## Discussion

From a sample of nearly 6000 nursing homes overseen by 5 CMS regional offices, we found that 1 of 10 facilities were at risk of exposure to severe hurricane-related inundation that would preclude the safe ground evacuation of residents and transport of essential medications and subsistence provisions. Across all regions, we observed multiple emergency preparedness deficiencies in domains that are critical to either safely evacuating residents or sheltering residents in place during severe weather events. We also found considerable geographic variation in the association between nursing home inundation exposure and the presence and number of critical emergency preparedness deficiencies. These findings suggest that nursing home emergency preparedness may not be consistently responsive to or commensurate with the environmental risks to which facilities are exposed.

Exposed facilities within the Mid-Atlantic region had a significantly greater likelihood of emergency preparedness deficiencies. Exposed facilities within the Southeast and Eastern Gulf Coast and New York metropolitan area exhibited directionally similar patterns, although the associations were lacking. Although we cannot exclude the possibility of state surveyors exhibiting greater vigilance based on perceived exposure risk within these regions, this possibility is lessened by the absence of a dose-response association between inundation severity and the likelihood or frequency of emergency preparedness deficiencies. If surveyors were uniquely sensitive to exposure severity in regions with a positive association between exposure and emergency preparedness deficiencies, one would expect to see the magnitude of these associations increase as the inundation threshold increased. Within the Western Gulf Coast (regional office 6), exposure status appeared to be protective against emergency preparedness deficiencies. New England facilities (regional office 1) demonstrated a similar pattern for the presence and number of critical emergency preparedness deficiencies, although these associations were not statistically significant. The most prevalent deficiencies are related to the ability of nursing homes to safely shelter in place or evacuate residents. Especially concerning is the high prevalence of inadequate subsistence provisions or standby power systems, as these factors may prompt unnecessary evacuations and thereby compound risks to vulnerable residents.^32,33^

Considering the differential susceptibility of nursing home residents to external stressors, emergency preparedness should be better aligned with the likelihood and severity of exposure to environmental hazards, including hurricane-related inundation. Projected increases in the intensity and geographic distribution of hurricanes within coastal regions further heighten the importance of this issue.^4,34^ The stark regional variation in emergency preparedness by exposed nursing facilities suggests that it may be useful to isolate and disseminate the strategies used by nursing home administrators, staff, and regulators within high-performing regions.^35^ Because Western Gulf Coast nursing homes exhibited emergency preparedness adherence that was well aligned with the local environmental risks (ie, exposed facilities were less likely to have critical emergency preparedness deficiencies than their unexposed counterparts), they could potentially serve as a model for other regions. Possible explanations for the risk-responsive emergency preparedness of nursing homes in the Western Gulf Coast may include reforms following their prior experience in hurricane response.^3,36^ For example, Gulf Coast nursing home residents incurred substantial morbidity and loss of life in the wake of Hurricanes Katrina and Rita.^36^ Ensuing investigations determined that inadequate emergency preparedness and inappropriate evacuation decisions contributed to these costs, prompting increased federal, regional, and state oversight.^3,7,37^

In addition to learning from high-performing regions, improved integration of nursing homes into local disaster planning agencies may better align emergency preparedness with the magnitude of environmental risks to which facilities are exposed. Based on our results, which support concerns that nursing homes may be poorly integrated into community disaster response models under the CMS all-hazards framework,^14,15^ we suggest that state survey agencies and CMS regional offices consider stronger oversight and enforcement of these partnerships. Because it would be impractical for CMS regional offices to appraise and monitor environmental risks, partnerships with local and regional emergency planning agencies should be relied upon to identify facilities exposed to salient regional and local environmental hazards. In turn, regulators could identify which facilities should be prioritized for oversight based upon their historical audit performance. A regulatory framework that is responsive to environmental hazards might involve a relatively greater inspection frequency for facilities that have outstanding critical emergency preparedness deficiencies and are at disproportionate risk of exposure to regionally concentrated environmental hazards (such as hurricanes along the Atlantic and Gulf Coasts).^15^ Although facilities meeting these criteria should receive more intensive oversight, these changes may produce a cost-effective and more efficient allocation of oversight responsibilities.^28,38,39^ These partnerships may also offer valuable insight as to the alignment between facilities’ exposure risk and their structural ability to withstand inundation, as determined by available building code guidance.^40^

### Limitations

This study has some limitations. Our ability to classify exposure is restricted to information aggregated within the NHC storm surge hazard maps. The developers of this resource note several potential limitations regarding the assumptions required to build this product. First, the data products do not account for increases in water levels due to waves, which may result in the underestimation of inundation by 10% to 50%.^19,41^ Second, physical processes affecting water levels outside of the areas for which the SLOSH models are validated cannot be modeled.^19^ Because the storm surge hazard maps represent maximum inundation scenarios, without probabilities corresponding to hurricane category, it would be desirable to repeat this analysis with a broader range of risk scenarios as such data products become available. We refrained from incorporating assumptions regarding the respective probabilities of Saffir-Simpson Hurricane Wind Scale categories to avoid compounding uncertainties across distinct climate models as risk projections continue to evolve.^42^ Third, the NHC data are constrained to hurricane-related inundation and may, therefore, underestimate inundation exposure due to factors including heavy rainfall.^43^ It would be instructive to replicate this analysis using geospatial data that reflect the risk of severe flooding due to multiple potential causes.

We cannot exclude the possibility that state surveyors were more vigilant in issuing emergency preparedness deficiencies based on perceived inundation risk. To address this limitation, we conducted sensitivity analyses that evaluated associations between emergency preparedness and increasingly severe exposure thresholds. Because regulators’ sensitivity to exposure increments has not been previously evaluated, we used a multiplicative scale to test the effect of large differences in inundation thresholds. Although the results of our additional analyses did not suggest a dose-response association between exposure severity and the likelihood of critical emergency preparedness deficiencies, they cannot rule out differences in the behavior of state surveyors or regional oversight practices.

Our evaluation assumes that administrative deficiencies are reasonable indicators of nursing homes’ emergency preparedness, but adherence to CMS emergency preparedness guidance does not necessarily ensure effective execution of organizational emergency planning or response.^14^ Nonetheless, the CMS standards subsume foundational competencies that are well aligned with national emergency preparedness guidance and prior literature.^7,24,25^ Moreover, inadequate emergency preparedness has contributed to adverse resident outcomes during prior emergencies due to severe weather events.^2^

Additionally, the observational nature of this study precludes our ability to isolate organizational or regulatory behaviors and practices that may underlie regional differences in the emergency preparedness of nursing homes at increased risk of exposure to hurricane-related inundation. Complementary qualitative analyses may help to elucidate the mechanisms underlying regional variation and identify practices that better align emergency preparedness and local environmental risk.

## Conclusions

Ensuring adequate nursing home emergency preparedness is vital to the safety and well-being of vulnerable older persons, especially as community exposure to hurricane episodes is projected to increase. We demonstrated considerable regional variability in the emergency preparedness of nursing homes exposed to hurricane-related inundation. We observed an association between emergency preparedness and potential inundation exposure for Mid-Atlantic nursing homes, suggesting that these exposed facilities with critical emergency preparedness deficiencies should be prioritized for remediation. Given their competing responsibilities and lack of formal training in this domain, nursing home administrators and staff may require additional support with their assessment and response to environmental risks under an all-hazards approach. Based on our findings, CMS regional offices may need to enhance enforcement of nursing home partnerships with local emergency planning agencies, which are better equipped to appraise and address environmental risks. Finally, the favorable preparedness of nursing homes within the Western Gulf Coast region suggests that this region could serve as an exemplar of risk-responsive emergency planning and oversight.

## Notes

Supplement 1. **eTable 1.** Frequency of CMS Deficiency Codes for the 5914 Atlantic and Gulf Coast Nursing Homes **eTable 2.** Prevalence of Exposed Facilities for Different Storm Surge Exposure Thresholds **eTable 3.** Prevalence of Most Common Emergency Preparedness Deficiencies by CMS Regional Office **eTable 4.** Associations Between Inundation Exposure and Emergency Preparedness Deficiencies for Alternative Exposure Definitions

Supplement 2.
Data Sharing Statement
