# Supplementary material for: Association of Nursing Home Exposure to Hurricane-Related Inundation With Emergency Preparedness
Source: JAMA Netw Open. 2023 Jan 6;6(1):e2249937. doi: 10.1001/jamanetworkopen.2022.49937 (PMC9856665; doi:10.1001/jamanetworkopen.2022.49937)
Supplement: Supplement 1. — eTable 1. Frequency of CMS Deficiency Codes for the 5914 Atlantic and Gulf Coast Nursing Homes eTable 2. Prevalence of Exposed Facilities for Different Storm Surge Exposure Thresholds eTable 3. Prevalence of Most Common Emergency Preparedness Deficiencies by CMS Regional Office eTable 4. Associations Between Inundation Exposure and Emergency Preparedness Deficiencies for Alternative Exposure Definitions [file jamanetwopen-e2249937-s001.pdf]

Supplementary Online Content

Festa N, Throgmorton KF, Heaphy N, Canavan M, Gil TM. Association of nursing home exposure to hurricane-related inundation with emergency preparedness. *JAMA Netw Open*. 2023;6(1):e2249937. doi:10.1001/jamanetworkopen.2022.49937

eTable 1. Frequency of CMS Deficiency Codes for the 5914 Atlantic and Gulf Coast Nursing Homes
eTable 2. Prevalence of Exposed Facilities for Different Storm Surge Exposure Thresholds
eTable 3. Prevalence of Most Common Emergency Preparedness Deficiencies by CMS Regional Office
eTable 4. Associations Between Inundation Exposure and Emergency Preparedness Deficiencies for Alternative Exposure Definitions

This supplementary material has been provided by the authors to give readers additional information about their work.

© 2023 Festa N et al. *JAMA Network Open*.

eTable 1. Frequency of CMS Deficiency Codes for the 5914 Atlantic and Gulf Coast Nursing Homes

Deficiency Code[a]	Description	Frequency	Percent (%)
E-0039	Conduct testing and exercise requirements	577	9.8
E-0015	Address subsistence needs for staff and patients	414	7.0
E-0037	Establish staff and initial training requirements	367	6.2
E-0026	Establish roles under a Waiver declared by secretary	364	6.2
E-0004	Develop and maintain an Emergency Preparedness Program (EP)	285	4.8
E-0024	Establish policies and procedures for volunteers	279	4.7
E-0036	Establish emergency prep training and testing	262	4.4
E-0006	Conduct risk assessment and an All-Hazards approach	249	4.2
E-0009	Include a process for Emergency Preparedness collaboration	248	4.2
E-0007	Address patient/client population and determine types of services needed	207	3.5
E-0035	Provide family notifications of emergency plan	203	3.4
E-0018	Establish procedures for tracking staff and patients during an emergency	174	2.9
E-0020	Establish policies and procedures including evacuation	171	2.9
E-0001	Establish an Emergency Preparedness Program (EP)	169	2.9
E-0041	Implement emergency and standby power systems	165	2.8
E-0023	Establish policies and procedures for medical documentation	150	2.5
E-0030	List the names and contact information of those in the facility	146	2.5
E-0013	Develop Emergency Preparedness policies and procedures	141	2.4
E-0029	Develop a communication plan	131	2.2
E-0034	Provide a means of sharing information on occupancy/needs	119	2.0
E-0022	Establish policies and procedures for sheltering	112	1.9
E-0031	Provide emergency officials' contact information	105	1.8
E-0032	Provide primary/alternate means for communication	97	1.6
E-0025	Create arrangements with other facilities to receive patients	88	1.5
E-0033	Establish methods for sharing information	70	1.2
K-048	A written emergency evacuation plan	0	0.0
K-050	Conduct routine fire drills	0	0.0

Abbreviation(s): CMS, Centers for Medicare & Medicaid Services
a: CMS Life Safety Code Emergency Preparedness Deficiency Tags

© 2023 Festa N et al. *JAMA Network Open*.

eTable 2. Prevalence of Exposed Facilities for Different Storm Surge Exposure Thresholds

**Regional Office**	**Total Facilities (N)**	**≥2-Feet**		**≥4-Feet**		**≥6-Feet**	
		n	%	n	%	n	%
New England	819	35	4.3	29	3.5	25	3.1
New York Metro Area	967	103	10.7	96	9.9	89	9.2
Mid-Atlantic	565	30	5.3	24	4.3	12	2.1
Southeast/Eastern Gulf	2,103	289	13.7	253	12.0	225	10.7
Western Gulf	1,460	160	11.0	144	9.9	124	8.5

Abbreviation(s): SD: standard deviation; CMS Centers for Medicare & Medicaid Services

© 2023 Festa N et al. *JAMA Network Open*.

eTable 3. Prevalence of Most Common Emergency Preparedness Deficiencies by CMS Regional Office

**Deficiency Code**[a]	Description	Frequency	Percent (%)
**New England (n=819)**			
E-0039	Conduct testing and exercise requirements	94	11.5
E-0037	Establish staff and initial training requirements	78	9.5
E-0035	Provide family notifications of emergency plan	57	7.0
E-0009	Include a process for Emergency Preparedness collaboration	52	6.4
E-0036	Establish emergency prep training and testing	51	6.2
**New York Metro Area (n=967)**			
E-0026	Establish roles under a Waiver declared by secretary	121	12.5
E-0039	Conduct testing and exercise requirements	83	8.6
E-0024	Establish policies and procedures for volunteers	83	8.6
E-0037	Establish staff and initial training requirements	81	8.4
E-0015	Address subsistence needs for staff and patients	63	6.5
**Mid-Atlantic (n=565)**			
E-0037	Establish staff and initial training requirements	41	7.3
E-0026	Establish roles under a Waiver declared by secretary	38	6.7
E-0036	Establish emergency prep training and testing	36	6.4
E-0035	Provide family notifications of emergency plan	34	6.0
E-0007	Address patient/client population and determine types of services needed	34	6.0
**Southeast/Eastern Gulf (n=2,103)**			
E-0015	Address subsistence needs for staff and patients	207	9.8
E-0039	Conduct testing and exercise requirements	106	5.0
E-0004	Develop and maintain an Emergency Preparedness Program (EP)	105	5.0
E-0026	Establish roles under a Waiver declared by secretary	93	4.4
E-0001	Establish an Emergency Preparedness Program (EP)	86	4.1
**Western Gulf (n=1,460)**			
E-0039	Conduct testing and exercise requirements	260	17.8
E-0037	Establish staff and initial training requirements	122	8.4
E-0009	Include a process for Emergency Preparedness collaboration	109	7.4
E-0004	Develop and maintain an Emergency Preparedness Program (EP)	97	6.6
E-0006	Conduct risk assessment using All-Hazards approach	96	6.6

Abbreviation(s): SD: standard deviation; CMS Centers for Medicare & Medicaid Services; RO, Regional Office
a: CMS Life Safety Code Emergency Preparedness Deficiency Tags

© 2023 Festa N et al. *JAMA Network Open*.

eTable 4. Associations Between Inundation Exposure and Emergency Preparedness Deficiencies for Alternative Exposure Definitions

	Dichotomous (Primary) Outcome		Count (Secondary) Outcome	
	≥4-Feet Inundation	≥6-Feet Inundation	≥4-Feet Inundation	≥6-Feet Inundation
	Odds Ratio, 95% CI [a]	Odds Ratio, 95% CI [a]	Rate Ratio, 95% CI [a]	Rate Ratio, 95% CI [a]
New England	0.51 (0.27-0.97)	0.82 (0.51-1.31)	0.90 (0.45-1.80)	1.05 (0.54-2.05)
New York Metro Area	1.29 (0.93-1.77)	1.27 (0.92-1.75)	1.25 (0.79-1.98)	1.25 (0.78-2.00)
Mid-Atlantic	1.68 (1.00-2.83)	0.50 (0.13-1.92)	2.49 (1.36-4.55)[†]	1.40 (0.40-4.94)
Southeast/Eastern Gulf	0.99 (0.82-1.21)	1.07 (0.89-1.29)	1.13 (0.89-1.43)	1.22 (0.97-1.54)
Western Gulf	0.82 (0.51-1.31)	0.82 (0.48-1.40)	0.63 (0.41-0.99)	0.59 (0.37-0.94)

Abbreviation(s): RO, Regional Office; CI, confidence interval

a: The reported associations are adjusted for rurality, proprietary ownership, facility size, Medicaid share, CMS 5-Star Quality and Staffing Ratings.
†: Estimates accompanied by a dagger symbol indicate a statistically significant association after Benjamin-Krieger-Yekutieli false discovery rate correction

© 2023 Festa N et al. *JAMA Network Open*.
