# Supplementary material for: Association of Nursing Home Exposure to Hurricane-Related Inundation With Emergency Preparedness
Source: JAMA Netw Open. 2023 Jan 6;6(1):e2249937. doi: 10.1001/jamanetworkopen.2022.49937 (PMC9856665; doi:10.1001/jamanetworkopen.2022.49937)
Supplement: Supplement 2. — Data Sharing Statement [file jamanetwopen-e2249937-s002.pdf]

Data Sharing Statement

Festa. Association of Nursing Home Exposure to Hurricane-Related Inundation With Emergency Preparedness. *JAMA Netw Open*. Published January 06, 2023. doi:10.1001/jamanetworkopen.2022.49937

Data
Data available: No

Additional Information
Explanation for why data not available: All data used for this study are publicly available with complete references to data sources included in the text.
